# HIV-Antiretroviral Therapy Induced Liver, Gastrointestinal, and Pancreatic Injury

**DOI:** 10.1155/2012/760706

**Published:** 2012-03-11

**Authors:** Manuela G. Neuman, Michelle Schneider, Radu M. Nanau, Charles Parry

**Affiliations:** ^1^Departments of Clinical Pharmacology and Toxicology, and Global Health, University of Toronto, ON, Canada M5G 1X8; ^2^In Vitro Drug Safety and Biotechnology, University of Toronto, MaRS Discovery District, 101 College Street, Suite 300, Lab 351, Toronto, ON, Canada M5G 1L7; ^3^Alcohol & Drug Abuse Research Unit, Medical Research Council, Tygerberg (Cape Town), South Africa; ^4^Department of Psychiatry, Stellenbosch University, Tygerberg (Cape Town), South Africa

## Abstract

The present paper describes possible connections between antiretroviral therapies (ARTs) used to treat human immunodeficiency virus (HIV) infection and adverse drug reactions (ADRs) encountered predominantly in the liver, including hypersensitivity syndrome reactions, as well as throughout the gastrointestinal system, including the pancreas. Highly active antiretroviral therapy (HAART) has a positive influence on the quality of life and longevity in HIV patients, substantially reducing morbidity and mortality in this population. However, HAART produces a spectrum of ADRs. Alcohol consumption can interact with HAART as well as other pharmaceutical agents used for the prevention of opportunistic infections such as pneumonia and tuberculosis. Other coinfections that occur in HIV, such as hepatitis viruses B or C, cytomegalovirus, or herpes simplex virus, further complicate the etiology of HAART-induced ADRs. The aspect of liver pathology including liver structure and function has received little attention and deserves further evaluation. The materials used provide a data-supported approach. They are based on systematic review and analysis of recently published world literature (MedLine search) and the experience of the authors in the specified topic. We conclude that therapeutic and drug monitoring of ART, using laboratory identification of phenotypic susceptibilities, drug interactions with other medications, drug interactions with herbal medicines, and alcohol intake might enable a safer use of this medication.

## 1. Introduction

Knowledge about indications for antiretroviral therapy (ART) use in chronically human immunodeficiency virus (HIV-) infected patients, relative efficacy of different regimens, patient evaluation, and laboratory monitoring are essential in the success of viral eradication. There are different combination therapies presenting activity against both wild-type and multidrug resistant HIV.

Side effects of these therapeutic interventions include adverse drug reactions (ADRs) such as direct hepatocytotoxicity, hypersensitivity syndrome reactions (HSRs), nausea, headache, diarrhea, and pancreatic toxicity. An ADR represents any noxious, unintended, and undesired effect of a drug, which occurs at doses used in humans for prophylaxis, diagnosis, or therapy [1].

Pharmaceutical agents that can be combined to make up highly active antiretroviral therapy (HAART) can be divided into three categories, namely, nucleoside reverse transcriptase inhibitors (NRTIs), nonnucleoside reverse transcriptase inhibitors (NNRTIs), and protease inhibitors (PIs), based on their mechanism of action. Substrates of P-glycoprotein, an ATP-dependent efflux membrane multidrug resistance transporter, comprise one class of molecules that can limit the absorption of most PIs. For example, oral administration of saquinavir, indinavir, or nelfinavir in knockout mice lacking this transporter resulted in two- to fivefold increases in plasma drug concentrations [2]. Higher plasma drug concentrations can therefore produce toxicities in human patients that might lack P-glycoprotein.

While drug interactions should be examined closely whenever prescribing medication in combination with PIs, this is a particularly important consideration with ritonavir, given its powerful inhibition of cytochrome p450 (CYP) 3A4 and its effects on several other mechanisms of drug interactions [3]. These can lead to increased levels of many coadministered medications, and consequently ADRs. Moreover, there is a potential for interaction with nutritional supplements [4].

Physicians should also be aware that patients with chronic viral hepatitis coinfection have additional impairment of CYP3A activity in the presence of ritonavir, compared to HIV patients without viral hepatitis, even at the low doses of 100 mg/day typically used for pharmacokinetic boosting [61].

The various ADRs associated with ART use encountered predominantly in the liver, including HSRs, as well as throughout the gastrointestinal (GI) system, including the pancreas, are presented hereinafter and summarized in Table 1.

## 2. Hepatotoxicity

Mendes-Corrêa et al. argue that liver damage exists in HIV patients independent of ART exposure [62].

In general, severe hepatic injury occurs in HAART patients, regardless of their treatment [63]. In his last published work, Zimmerman stated unequivocally that the necroinflammatory changes that can be seen in drug-related hepatotoxicity can overlap with those of chronic viral hepatitis [64].

The importance of histological changes in the diagnosis of drug-induced toxicity, its disease spectrum, and the fine structures of hepatocytotoxicity are considered in the discussion section. In the present section, we bring forth only evidence shown by investigators in their work, which is also summarized in Table 1.

Careful review of medication, both prescription and nonprescription, should be compiled in patients with new symptoms or signs of hepatitis, in order to address the possibility of drug toxicity.

Hepatic mitochondrial damage was found in ART-naive patients as well as patients exposed to the NRTIs zidovudine or didanosine [62]. The intensity of dense granules was higher in mitochondria from previously untreated patients, compared to current ART patients (*P* < 0.05). Qualitative analyses showed areas of mitochondrial hyperplasia, with changes in shape (elongation, baloonization, bizarre shapes) and size (megamitochondria) in both groups. There were also increases in the numbers of dense granules, matrix condensation, crista loss, lamellar distributed filamentous material, and crystalloid material [62].

The levels of ^13^C-methionine exhaled, a measure of hepatic mitochondrial function, increased significantly in ART-naive patients after treatment initiation (*P* < 0.001) [5]. ^13^C exhalation continued to decrease in ART-naive patients who continued to remain naive (*P* = 0.04), as well as patients who stopped treatment (*P* = 0.043). No changes in the ^13^C-methionine breath test results were observed among ART-experienced patients who did not change their treatment (*P* = 0.31) or changed only the PI and NNRTI components of their treatments (*P* = 0.34), or among patients who remained on structured treatment interruption (*P* = 0.068). Reinitiation of ART led to significant improvements (*P* = 0.008) [5]. A switch from didanosine or stavudine to tenofovir or abacavir also led to a significant improvement in ^13^C-methionine breath test performance (*P* < 0.001) [5].

Hepatotoxicity is a relatively common ADR leading to treatment interruptions in HIV patients, observed with different drug combinations (Table 1) [6–27]. Among these, nevirapine was often associated with the development of hepatotoxicity [8, 9, 12, 13, 15, 16, 18, 20, 22, 23, 25]. Nevirapine use was associated with a higher incidence of liver toxicity than efavirenz use [14]. The use of PIs in combination with either efavirenz or nevirapine was associated with an increased risk of hepatotoxicity compared to efavirenz or nevirapine alone (odds ratio (OR) 3.07, 95% confidence interval (CI) 1.01–9.32, *P* = 0.04) [65].

Increases in liver enzymes are also common ADRs characteristic of different ART regimens (Table 1) [20, 28–35, 66]. Increases in alanine aminotransferase (ALT) or/and aspartate aminotransferase (AST) are common symptoms of hepatotoxicity, while increases in alkaline phosphatase and *γ*-glutamyl transpeptidase were indicative of cholestasis in one study [66].

The median delay between HAART initiation and occurrence of hepatotoxicity was 2.5 months (interquartile range (IQR) 1 to 11 months) in one study [17] and 5 weeks (IQR 3 to 29 weeks) in another study [18]. While a similar number of patients discontinued nevirapine due to hepatotoxicity before month 3 and after a mean number of 9 months in another study [13], van Griensven et al. observed that only 27.6% of 29 cases of nevirapine hepatotoxicity occurred after 6 months of treatment [18].

One study found hepatitis to occur with a similar frequency among zidovudine/lamivudine, zidovudine/didanosine, or stavudine/lamivudine patients [27], whereas a separate study found a higher incidence of hepatotoxicity among stavudine/lamivudine patients [19].

Hepatic events were the most common drug ADRs associated with atazanavir/ritonavir [24]. Jaundice was also observed among atazanavir/ritonavir patients [24, 33, 36], but not among lopinavir/ritonavir patients [33]. Similarly, grade ≥3 increases in total bilirubin levels occurred more frequently in the atazanavir/ritonavir group than in the lopinavir/ritonavir group [33]. Acute liver failure, accompanied by jaundice, fever, vomiting, and hepatomegaly, was observed in a 10-year-old male [67]. The patient's condition started improving following liver transplantation and replacement of efavirenz with raltegravir [67]. Bilirubin levels did not affect the rate of hepatotoxicity in another study [35]. Grade 3 hyperbilirubinemia/liver toxicity was also observed with nevirapine [21].

Unconjugated bilirubin levels should be monitored in PI patients. The microsomal enzyme uridine diphosphate glucuronosyltransferase (UGT) mediates conjugation of bilirubin.

Hyperbilirubinemia is an adverse effect that occurs in approximately 25% of indinavir patients, with total bilirubin rises to the 2.5 to 5 mg/dL range [68]. This represents largely indirect bilirubin and is insignificant except as a possible complication of pregnancy [68, 69]. Atazanavir also appears to impair UGT activity [70], such that the PIs atazanavir and indinavir are associated with hyperbilirubinemia. The relationship between underlying genetic risk factors and the risk of developing hyperbilirubinemia remains unclear. UGT1A1*28 allele was associated with jaundice in a study in which bilirubin levels were not measured [24]. In a study of patients who underwent genotypic analysis for polymorphisms associated with increased unconjugated bilirubin, 64 (66.7%) of 96 patients were positive for the UGT1A1*28 allele. [70]. Ocama et al. found that 23 (29.9%) of 77 consecutive HIV-infected patients presenting with hepatotoxicity (jaundice, right upper quadrant pain with fever or malaise, ascites, and/or tender hepatomegaly) had increased transaminase levels as a result of nevirapine and/or isoniazid hepatotoxicity [71]. Of these 23 patients with drug-induced liver disease, 14 (60.9%) presented with jaundice and recovered after drug discontinuation. Hepatitis B surface antigen was positive in 11 (14.3%) patients while antihepatitis C antibody was reactive in only 2 (2.6%). Granulomatous hepatitis due to tuberculosis was diagnosed in 7 (9.1%) patients. Other diagnoses included alcoholic liver disease, AIDS cholangiopathy, hepatocellular carcinoma, schistosomiasis, hemangioma, and hepatic adenoma. Twelve (15.6%) patients died during follow-up, of which 7 (9.1%) died because of liver disease [71].

The overall incidence of severe hepatic injury was not significantly different between NRTIs, NNRTIs, and PIs in a sample of 222 patients, of which 84 (37.8%) were coinfected with hepatitis C virus (HCV) [63]. Coinfection with hepatitis viruses is often associated with a higher risk of hepatotoxicity [63, 65]; however this is not always the case [24]. Elevated baseline liver function tests and older age are additional risk factors for hepatotoxicity [18].

## 3. Hypersensitivity Syndrome Reaction

HSRs have been associated with the NRTI abacavir, the NNRTIs nevirapine and efavirenz, and the PI amprenavir [72–74]. The potential for HSR development symbolizes a treatment-limiting and potentially life-threatening ADR.

Abacavir HSR is the major treatment-limiting toxicity of HAART regimens containing this drug. This ADR usually occurs in the first 6 weeks of treatment [75]. An HSR characterized by some combination of flu-like symptoms, fever, rash, as well as GI symptoms, including hepatotoxicity, generally occurs in 3–5% of patients starting abacavir [76]. Other symptoms of HSR include malaise, lethargy, myalgia, myolysis, arthralgia, edema, pharyngitis, cough, dyspnea, headache, and paresthesia. Physical findings may include lymphadenopathy, mucous membrane lesions (i.e., conjunctivitis, mouth ulcerations), and rash, which usually appears as maculopapular or urticarial, but can also lead to Stevens-Johnson syndrome (SJS).

Differentiation between abacavir HSR and viral respiratory infections can be problematic. Rash (OR 13.1, *P* = 0.02), nausea (OR 30, *P* < 0.001), vomiting (OR 17.1, *P* = 0.001), and diarrhea (OR 22, *P* < 0.001) were associated with HSR in 15 cases of abacavir HSR matched with 30 controls with culture-proven influenza A with no abacavir exposure [77]. The number of GI symptoms was also predictive of HSR (*P* < 0.001). Multivariate analysis confirmed that the number of GI symptoms (OR 8.6, *P* = 0.0032) and rash (OR 16.9, *P* = 0.07) was associated with abacavir HSR. Abacavir HSR-associated rash was typically mild to moderate in this study, occurring after an average of 9–11 days since treatment initiation [77]. Abacavir HSR was found to resolve itself rapidly following treatment modifications [37]. This reaction was observed in other studies as well [36–41].

Abacavir HSR is strongly associated with GI symptoms [77]. Laboratory abnormalities include elevated liver function tests, increased creatine phosphokinase or creatinine, and lymphopenia. Liver failure and death have occurred in association with HSR. Symptoms associated with HSR worsen with continued therapy but often resolve upon discontinuation of the drug [78, 79].

It is highly recommended that HSR patients avoid rechallenge with full-dose abacavir, as extremely severe symptoms and even death may result [80]. Reports describe the mechanisms of action, efficacy, and ADRs of abacavir in HIV-1-infected patients and illustrate the danger of serially rechallenging patients with this agent even if the patient was previously desensitized [78, 81–83].

A strong statistical association was identified between the human leukocyte antigen (HLA)-B*5701 allele, part of the major histocompatibility complex, and clinically diagnosed abacavir HSR [84]. While abacavir HSR occurs in approximately 5% of HIV patients treated with this drug, the HLA-B*5701 allele was discovered in 6.3% of 11000 genetic screens performed in a Canadian population [75]. As a consequence, prospective HLA-B*5701 screening is performed to identify patients at high risk for abacavir HSR before they are treated [85]. Genetic screening of potential abacavir patients can greatly help prevent HSRs and it can lead to individualizing of HAART in order to prevent toxicity and to improve adherence [75]. Carriers of HLA-B*5701 should avoid abacavir-based HAART [84, 86–91]. Despite prior HLA genotyping, the incidence of abacavir HSR was higher in an abacavir/lamivudine-based regimen compared to a tenofovir/emtricitabine-based regimen [40, 41]. This phenomenon indicates that an additional metabolic or immune mechanism might contribute to the ADR.

Approximately 17% of patients starting nevirapine and 10% of patients starting efavirenz will develop rash of varying severity with or without systemic features, typically between 1 and 3 weeks after starting the drug [92]. As HLA genotyping has the potential to reduce the incidence of abacavir HSR, nevirapine is the pharmaceutical agent most often associated with cutaneous HSRs (Table 1) [9–14, 16–18, 20–25, 31, 39, 42–48]. Moreover, Warren et al. [93] report that nevirapine can be associated with SJS. Albeit infrequent, SJS and toxic epidermal necrolysis (TEN) are sometimes observed in conjunction with nevirapine and can be fatal [9, 13, 31, 45, 48, 49]. The median time for skin rash occurrence was 1.0 month (IQR 3 weeks to 3 months) [17, 18, 45]. The majority of patients who discontinued nevirapine due to HSR did so within 18 weeks (for both skin rash and hepatotoxicity without concomitant skin rash) [47].

Hepatitis is observed with relative frequency in HSR patients [15, 45, 48]. No patient suffered from both skin rash and liver abnormality in other studies [8, 11, 44, 47]. Hepatic involvement in HSR can also be observed without concomitant cutaneous reactions [44, 47].

Nevirapine-associated HSRs are usually moderate to severe and often require treatment change for the elimination of the agent that caused the reaction [8, 9, 13, 15, 16, 18, 20, 23, 31, 42, 44–46]. Nevirapine is usually substituted with efavirenz in HSR cases [42]. Dermal lesions were observed only in combinations that contained nevirapine and lamivudine in one study [66]. There was a higher incidence of severe rashes (grade ≥3) among nevirapine patients, compared to nonnevirapine patients (*P* = 0.002) in another study. While the same trend was observed overall for grade ≥2 rash, this association was no longer significant (*P* = 0.099) [31]. Efavirenz itself has been associated with skin symptoms [20, 50]. In such cases, a switch to nevirapine often results in the development of similar reactions on nevirapine as well, showing cross-reactivity between the two NNRTIs [20]. Efavirenz treatment did not lead to the development of cutaneous HSRs in a separate study [25].

The HLA-DRB1*01 allele was significantly associated with isolated rash alone in patients exposed to nevirapine or efavirenz (*P* = 0.04), whereas immunologic and genetic factors are associated with hepatotoxicity and systemic ADRs [51].

Lopinavir/ritonavir [50] and atazanavir/ritonavir [24] were also associated with HSR. Among other NRTIs, the development of rash led to zidovudine and stavudine substitution [30] and the development of pruritis led to didanosine substitution [35] in other studies.

There are also studies in which the drugs responsible for the HSR are not specified, but certain hypotheses can be made based on the medication regimen. In such instances, patients are often exposed to either nevirapine or efavirenz [19, 52].

Older age (*P* < 0.003) and a higher CD4^+^ cell count (*P* < 0.03) were predictors of rash development [42]. No significant differences in plasma nevirapine concentrations were observed between patients who experienced skin rash and patients who did not [14]; however significantly more cases of grade ≤2 rash were identified in a group receiving a full dose of nevirapine, compared to a half-dose of the drug (*P* = 0.003) [42]. A strong association between grade ≥2 rash and nevirapine-based treatment was observed when only subjects with CD4^+^ >250 cells/mm^3^ were considered (*P* = 0.001), suggesting an interaction between the treatment and the CD4^+^ count [42].

In addition, a trend of increasing risk of developing grade ≥2 rash was observed in pregnant subjects (*P* = 0.054). Pregnant subjects with baseline CD4^+^ >250 cells/mm^3^ were significantly at risk of developing grade ≥2 rash (*P* = 0.042). However, pregnancy alone is not a predictor of ADR development for women initiating nevirapine therapy. This is an important finding, as pregnant women were both more likely to start nevirapine-based treatment (*P* < 0.001) and to have higher baseline CD4^+^ counts (*P* < 0.001) [31]. No independent risk factors for skin rash were identified in a separate study [18].

## 4. Gastrointestinal Intolerance

GI complaints, mainly diarrhea, vomiting, and abdominal disturbances, were the most frequently observed ADRs in several studies [24, 53]. These types of ADRs appeared mainly during the first 12 weeks of therapy and were mild (grade ≤2) and transient in most patients [53]. Gastroenterological intolerance (dyspepsia, nausea, vomiting, and diarrhea) is common effects of different drug combinations [66]. GI intolerance was the main cause of lopinavir/ritonavir therapy modification or interruption (Table 1) [24, 32, 33, 36, 54]. GI symptoms associated with lopinavir/ritonavir and tipranavir were the most common type of ADRs in patients exposed to these pharmaceutical agents [55]. Cases of GI toxicity associated with lopinavir/ritonavir discontinuation occurred between day 3 and week 15 [56]. While diarrhea was the most common ADR that led to lopinavir/ritonavir treatment changes, this ADR was less commonly associated with efavirenz discontinuations [26]. Compared with patients assigned to efavirenz, patients assigned to lopinavir/ritonavir had higher rates of nausea, diarrhea, and vomiting (*P* < 0.01) [57].

Nevirapine is another drug associated with a high rate of treatment discontinuations as result of GI intolerance [22, 23, 47]. Nevirapine discontinuations caused by GI symptoms often occur within the first 18 weeks of treatment [47].

The incidence of GI ADRs (mainly diarrhea) was higher in patients treated with nelfinavir compared to patients treated with nevirapine (*P* = 0.01) [22]. Vomiting and diarrhea were observed in other samples of patients treated with nelfinavir [28, 56].

GI intolerance was the main cause of saquinavir therapy modification or interruption as well [36]. A higher saquinavir C_min_ was associated with a higher incidence of serious GI ADRs [58]. In addition, higher saquinavir C_min_ was more prevalent in individuals with grade ≥3 GI side effects, compared with individuals with grade ≤2 GI side effects (*P* = 0.028) [58]. Mild nausea and diarrhea were also observed among saquinavir patients [59].

No patient on atazanavir/ritonavir discontinued treatment due to GI intolerance. More patients receiving lopinavir/ritonavir experienced grade ≥2 nausea, compared to patients receiving atazanavir/ritonavir [33].

GI symptoms were associated with treatment modifications in patients receiving treatment with dual-boosted PIs [94]. The drugs responsible for the observed ADRs are not specified [94]. GI toxicity was also reported in relation to didanosine [34]. Patients assigned to zidovudine and didanosine had higher rates of nausea, constipation, and fatigue when compared to patients assigned to stavudine and lamivudine (*P* < 0.05) [57]. Drug-related GI toxicity leads to poor medication adherence and ultimate virological failure [34]. Mild GI intolerance that did not require treatment modifications was observed in a couple of other studies [46, 95].

## 5. Pancreatic Toxicity

Acute pancreatitis is an inflammatory condition of the pancreas characterized clinically by abdominal pain and elevated levels of pancreatic enzymes (serum amylase, isoamylase, and/or lipase). Abnormal exocrine and endocrine function can also occur during an acute attack.

Banks and Freeman argue that acute pancreatitis is characterized by two of either abdominal pain characteristic of acute pancreatitis, serum amylase, and/or lipase raised ≥3 times over the upper limit of normal, and characteristic findings of acute pancreatitis on computed tomography (CT) scan [96]. Based on this characterization, Manfredi and Calza found 46 (3.7%) patients who presented with serum amylase and/or lipase raised ≥3 times over the upper limit of normal and acute pancreatitis on CT scan [60]. A further 120 (11.1%) patients presented only with serum amylase and/or lipase raised ≥3 times over the upper limit of normal and were thus classified as asymptomatic. Only 31 (2.9%) patients had mild-to-moderate symptoms of abdominal pain, with only 9 cases of clinically assessed pancreatits, none of which required surgery or developed complications [60].

A relatively high incidence of at least one confirmed laboratory pancreatic abnormality, relating to at least two serum pancreatic enzymes over a mean follow-up period of 33.6 consecutive months, was observed in this large study [60]. The use of NRTIs like didanosine, stavudine, and lamivudine and coadministration of other medications such as pentamidine, cotrimoxazole, antituberculosis therapy, cytotoxic chemotherapy, or their combination for at least 6 months were significant risk factors for at least 3-fold increases in serum pancreatic enzymes (*P* < 0.05), as was drug or alcohol abuse for at least 6 months (*P* = 0.04). Opportunistic infections with potential pancreatic involvement (cytomegalovirus, cryptosporidiosis, mycobacteriosis, or disseminated tuberculosis) (*P* = 0.03), chronic liver, and/or biliary disease (*P* = 0.01), current administration of HAART regimen containing PIs (*P* = 0.05), hypertriglyceridemia for at least 6 months (*P* = 0.02), or a combination of the above risk factors (*P* = 0.003) were also associated with pancreatic toxicity [60]. The combination of hyperamylasemia with either elevated isoamylasemia or lipasemia was selected for evaluating laboratory abnormalities, and the authors do not separate hyperamylasemia from hyperlipasemia. Serum isoamylase and serum lipase measurements are more specific when compared with serum amylase alone for the diagnosis of pancreatitis [60]. Even so, Van Dyke et al. chose to diagnose pancreatitis based on total serum amylase rather than the more specific pancreatic serum amylase, as the former is routinely monitored [27]. The PI lopinavir/ritonavir was associated with amylase elevations in another study [32].

Pancreatitis likely attributable to didanosine was observed in a couple of studies (Table 1) [27, 35]. Grade ≥3 serum amylase elevations were similar in patients receiving either didanosine/lamivudine/efavirenz or lamivudine/zidovudine/efavirenz [97]. Hyperamylasemia and hyperuricemia were eventual findings without clinical relevance in another study [66]. Among NNRTIs, nevirapine was associated with pancreas-related toxicities [25, 47, 48], whereas efavirenz was not [25].

Recurrent episodes of acute pancreatitis may also suggest a misuse of alcohol or use of concomitant medication. There is no mention of how many drinkers were in a large study, yet the incidence of symptomatic and asymptomatic cases was similar between alcohol drinkers and abstainers [60].

## 6. HAART Interaction with Alcohol Consumption

Hepatic injury is often more common in individuals with alcohol abuse and in those with HCV coinfection. HAART-induced hepatic injury has the potential to limit the usefulness of this medication in HIV treatment [63]. Twelve (5.4%) patients were found to abuse alcohol in a sample of 222 patients, of which 84 (37.8%) were coinfected with HCV. Alcohol abuse was identified as a risk factor for developing hepatic injury of any grade (OR 3.42, 95% CI 1.04–11.19, *P* < 0.05), especially severe hepatic injury (OR 8.66, 95% CI 2.47–30.40, *P* < 0.05), measured by elevations in transaminase levels [63]. Alcohol intake greater than 40 g per day (OR 3.09, 95% CI 1.27–7.54, *P* = 0.01) was associated with a greater risk of severe hepatotoxicity in a sample of 108 patients [65].

Fourteen (10.6%) patients were alcohol abusers in a small sample of 132 HIV patients coinfected with HCV. Due to the low number of alcohol abusers in this sample, no association between alcohol abuse and hepatotoxicity was observed [98]. Alcohol consumption, both at baseline and during follow-up, was not linked to progression of fibrosis by ≥1 stages among 135 patients coinfected with HCV, of which 31 (23.0%) patients had an alcohol intake of >50 g/day [99].

Excessive alcohol consumption had no effect on the development of mild-to-moderate rash. However, severe rash plus/or hepatotoxicity was observed among 741 patients, of which 163 (22.0%) abused alcohol (≥168 g of alcohol per week for women and ≥252 g of alcohol per week for men) [16].

Since the primary metabolic pathways of abacavir are mediated by microsomal UDP glucuronyl transferase and cytosolic alcohol dehydrogenase, use and misuse of alcohol can lead to hepatotoxicity. A significant pharmacokinetic interaction was found following the coadministration of abacavir and ethanol. Twenty-four HIV-positive men received either a single 600 mg dose of abacavir, 0.7 g/kg ethanol (the equivalent of 5 alcoholic drinks), or a 600 mg dose of abacavir plus 0.7 g/kg ethanol on separate occasions. With coadministration, there was a 41% increase in abacavir area under the curve and a 26% increase in abacavir *t*_1/2_, with no change in the pharmacokinetic profile of ethanol [100].

While not all studies found an association between alcohol consumption and a greater risk of HAART toxicity, studies where such parameters are investigated often use small population sizes, with a low proportion of alcohol abusers, making it difficult to uncover interactions.

## 7. Discussion

The present paper discusses hepatic, GI, and pancreatic ADRs related to various ART drugs and drug combinations. We also introduce a section on HSR, since HSRs encompass many of the clinical entities of hepatic and GI representations. We further describe some of the interactions between ART and other drugs and alcohol. Moreover, we briefly explore the influence of certain comorbidities, such as viral hepatitis, on ART-induced hepatotoxicity.

As with all ART medications, many clinically significant interactions are possible with PIs. For example, atazanavir cross-reacts with nevirapine. Atazanavir exposure is significantly lower when combined with this drug, and the risk of nevirapine toxicity may increase due to increased nevirapine exposure. In addition, atazanavir in combination with efavirenz is not recommended in treatment-experienced patients, since efavirenz significantly lowers atazanavir exposure. Concomitant didanosine/lamivudine exposure is not recommended in ART-naive patients receiving unboosted atazanavir due to potential toxicities. In addition, the virological response to abacavir may be diminished significantly by multiple NRTI-associated mutations and/or by reductions in phenotypic susceptibility to abacavir. However, many subjects showing evidence of baseline resistance to NRTIs respond to abacavir.

Of particular relevance to the HIV-infected population is coinfection with HCV. Hepatitis with aminotransferase elevations was reported, and it should be appropriately monitored [101].

Hepatocytotoxicity, or drug-induced liver injury, can be classified based upon clinical presentation and laboratory features, the mechanism of toxicity, and/or histological findings. The presence of serum bilirubin raised >3 times over the upper limit of normal along with aminotransferase elevations is associated with a more drastic prognosis than isolated aminotransferase abnormalities [102], an observation known as Hy's Law [103].

In addition to these acute hepatic presentations, some drugs are associated with chronic histological inflammatory changes and a clinical syndrome resembling autoimmune hepatitis, while others cause endothelial damage or thrombosis, leading to vascular complications such as venoocclusive disease [104]. Withdrawal of the offending drug usually leads to reversal of the injury. The patterns of acute injury may present as hepatocellular (cytotoxic) damage, cholestasis, a mixed pattern of cytotoxic and cholestatic injury, or, less commonly, steatosis [102]. Discontinuation of the offending agent usually results in complete recovery, although the prognosis is generally worse in patients with hepatocellular injury presenting with jaundice when compared to cytotoxic injury alone.

HIV *per se* may influence the ultrastructural architecture of the liver. In the liver of a patient living with AIDS, Phillips et al. found tubular structures mainly in the cytoplasm of endothelial cells and less frequently in Kupffer cells, macrophages, fibroblasts, and biliary cells. These changes were associated with the endoplasmic reticulum, representing a cellular response to virus-induced injury [105].

Drug-induced steatohepatitis may also resemble alcoholic liver disease [106, 107]. In addition, ethnicity plays a role in antiretroviral-induced toxicity [108].

Some of the subjects living with HIV that were included in these studies have a history or past or present alcohol consumption. From a histological point of view, alcoholic hepatitis presents enlargement of hepatocytes that may increase the vascular pressure in the acinus [109]. Mitochondrial changes, including megamitochodria or irregular mitochondria, as well as Mallory bodies, are also encountered. Mallory bodies (alcoholic hyalin) correspond to cytokeratine conglomerations of proteins that form filaments.

The predominant cell in the liver is the hepatocyte, which contains abundant cytoplasm. There are little amounts of carbohydrates and phospholipids in filaments seen in hepatocytes. These cytokeratine filaments represent an abnormal expression of the cytoskeleton. Ultrastructurally, irregular inclusions, which range from small conglomerates of filaments to large inclusions, occupy most of the cytoplasm [105]. A wide range of other ultrastructural changes in alcoholic liver disease can be seen in conjunction with HAART, such as cell necrosis, increased peroxisome numbers, and crystalloid inclusions. Also, there are infrequent bile duct proliferation and ground glass cytoplasmic inclusions that can be resolved after alcohol abstinence.

The genetic value of UGT in PI-induced hyperbilirubinemia is further discussed [110]. Rotger et al. showed that individuals homozygous for the A(TA)_7_TAA allele of UGT1A1*28 enzyme receiving atazanavir or indinavir were at increased risk of experiencing hyperbilirubinemia in the jaundice ranges. They studied in parallel a group of patients that have not been genotyped for UGT1A1 allele before prescribing atazanavir or indinavir as first-line agents versus patients that have been genotyped for UGT1A1*28. The “genotype-guided ART” narrowed the use of atazanavir or indinavir to individuals without the UGT1A1*28 allele. The authors conclude that genetic screening would lead to a theoretical 75% reduction in the incidence of hyperbilirubinemia in the jaundice range. The high incidence of the UGT1A1*28 allele might lead to high risk of developing jaundice in the setting of Gilbert syndrome when exposed to specific PIs [110]. UGT1A1 promoter A(TA)_7_TAA variant was most common among African Americans and least common among subjects of Asian origin [111]. Therefore the use of genetic screening for the A(TA)_7_TAA allele before initiation of antiretroviral therapy is controversial [112].

Ideally, genetic testing for this allele, in conjunction with testing for markers of immunotoxicity such as lymphocyte toxicity assay, may be used in the future in the clinical setting to prevent, diagnose, or monitor drug-induced ADRs in people living with HIV [113].

We conclude that antiviral pharmacodynamics is affected by a broad array of factors ranging from individual pharmacokinetic and pharmacogenetic parameters, to medication adherence and drug-drug interactions. Therefore, therapeutic and drug monitoring of HAART plays an important role. Using laboratory techniques to identify phenotypic susceptibilities, as well as knowing the interactions between ART and other drugs or herbal medicines, might enable a safer use of this beneficial type of medication in HIV patients. Adding to the complexity, many HIV-infected patients are unable to keep therapeutic medication safe due to their behavior patterns, such as alcohol misuse. Lack of pharmacovigilance is associated with HIV disease progression as well as toxicities.

The major objective of this article is to increase awareness on the possible toxicity of therapeutics prescribed in HIV. Moreover, there are other health products including traditional small molecule drugs, natural health products, biologics, and biotechnology products that are prescribed in HIV. These products may cause not only significant liver direct toxicity but also unpredictable, idiosyncratic hepatotoxicity. Therefore, in the process of achieving pharmacovigilance objectives, the investigational approach used for a particular therapeutic may have to be individualized based on the safety characteristics of the product as well as its proposed clinical application.

## Figures and Tables

**Table 1:** 

Study population	Study settings	Treatment	ADRs	Incidence of ADRs	Drugs associated with ADRs	Ref. no.
113 patients, 97 (85.8%) men	Germany	Stavudine/didanosine-based (*n* = 73 (64.6%))	Hepatic mitochondrial toxicity		Stavudine and didanosine	[5]

122 patients, 90 (73.8%) men	Germany	Zidovudine, lamivudine, abacavir, tenofovir	Hepatotoxicity	1 (0.8%) case	Tenofovir	[6]

600 adults, 430 (71.7%) women	Uganda	Zidovudine, lamivudine, abacavir (*n* = 300 (50.0%)) Zidovudine, lamivudine, nevirapine (*n* = 300 (50.0%))	Grade 4 liver function test abnormalities (ALT or/and AST raised >10 times over the upper limit of normal), including acute hepatitis/hepatic failure		Zidovudine Lamivudine Nevirapine	[7]

133 pregnant women	Brazil	Nevirapine-based	Grade ≤3 hepatotoxicity (ALT or/and AST raised ≤5 times over the upper limit of normal)	6 (4.5%) cases	Nevirapine	[8]
			Skin rash	21 (15.8%) cases	Nevirapine	

409 patients 244 (59.6%) pregnant women 87 (21.3%) nonpregnant women 78 (19.1%) men	Thailand	Zidovudine, lamivudine, nevirapine Stavudine, lamivudine, nevirapine	Hepatotoxicity, including asymptomatic hepatitis, symptomatic hepatitis or hepatitis occurring concomitant with rash	64 (15.6%) cases, including 19 (4.6%) symptomatic cases	Likely nevirapine	[9]
			Rash	66 (16.1%) cases, including 21 (5.1%) grade 4 cases	Nevirapine	
			SJS	6 (1.5%) cases	Nevirapine	

142 patients: 105 (73.9%) men, 124 (87.3%) white	Spain	Nevirapine-based Zidovudine, lamivudine (*n* = 65 (45.8%)) Stavudine, lamivudine (*n* = 34 (23.9%)) Stavudine, didanosine (*n* = 33 (23.2%)) Other combinations (*n* = 10 (7.0%))	Hepatotoxicity (ALT or/and AST raised >5 times over the upper limit of normal) Clinical hepatitis (ALT or/and AST raised >5 times over the upper limit of normal and/or nausea, asthenia, jaundice)	8 (5.63%) cases	Nevirapine	[10]
			Skin rash	16 (11.27%) cases	Nevirapine	

157 pregnant women	Brazil	Zidovudine, lamivudine, nevirapine	Grade ≤3 hepatotoxicity	7 (4.4%) cases	Nevirapine	[11]
			Skin rash	24 (15.3%) cases	Nevirapine	

540 patients	Niger	Stavudine, lamivudine, nevirapine	Grade ≤3 hepatotoxicity (ALT or/and AST raised ≤5 times over the upper limit of normal)	78 (15.7%) cases, including 6 (1.2%) grade 3 cases	Nevirapine	[12]
			Cutaneous ADRs	7 (1.3%) cases, including 5 (0.9%) rash cases and 2 (0.4%) pruritus cases	Nevirapine	

315 children median age 7.2 years: 158 (50.2%) girls	Rwanda	Stavudine, lamivudine, nevirapine (*n* = 281 (89.2%)). Stavudine, lamivudine, efavirenz (*n* = 6 (1.9%)) Zidovudine, lamivudine, nevirapine (*n* = 19 (6.0%)). Zidovudine, lamivudine, efavirenz (*n* = 9 (2.9%))	Severe hepatotoxicity, including asymptomatic hepatitis, symptomatic hepatitis, and clinical hepatitis	5 (1.7% of 300 nevirapine patients) cases before month 3, including 4 clinical hepatitis cases and 1 grade 3 asymptomatic case 3 (1.0% of 300 nevirapine patients) symptomatic hepatitis cases after a mean 9 months	Nevirapine	[13]
			Grade ≥3 skin manifestations, including SJS	16 (5.3% of 300 nevirapine patients) cases, including 2 (0.7%) SJS cases	Nevirapine	

8736 patient records	Tanzania	Various combinations	Liver toxicity		Nevirapine Efavirenz	[14]
			Skin rash	Higher in nevirapine patients than in efavirenz patients	Nevirapine Efavirenz	

253 women, 42 (16.6%) pregnant	United States	Nevirapine-based Zidovudine/lamivudine most common NRTI backbone	Hepatitis, including late-onset hepatitis	3 (1.2%) cases	Nevirapine	[15]
			Rash with concomitant hepatitis	2 (0.8%) cases	Nevirapine	
			Grade 1 rash	1 (0.4%) case	Nevirapine	

1110 patients, 631 (56.8%) men	Argentina	Nevirapine-based	Hepatotoxicity (ALT or/and AST raised >5 times over the upper limit of normal for patients with previously normal levels or >3.5 times over the baseline level for patients with abnormal basal levels)	35 (3.2%) cases	Nevirapine	[16]
			Severe rash	49 (4.4%) cases	Nevirapine	

290 women, 125 (43.1%) pregnant	Côte d'Ivoire	Zidovudine, lamivudine, nevirapine (*n* = 265 (91.4%)). Stavudine, lamivudine, nevirapine (*n* = 25 (8.6%))	Hepatotoxicity (ALT or/and AST raised >5 times over the upper limit of normal)	10 (3.4%) cases	Not specified	[17]
			Rash	15 (5.2%) cases	Likely nevirapine	

2190 adults, 1567 (71.5%) women	Rwanda	Stavudine, lamivudine, nevirapine	Hepatotoxicity (assessed based on ALT levels)	29 (1.3% of entire sample and 21.0% of 138 patients who stopped treatment due to nevirapine toxicity) cases	Nevirapine	[18]
			Skin rash	108 (4.9% of entire sample and 78.3% of 138 patients who stopped treatment due to nevirapine toxicity) cases	Nevirapine	

546 patients, 378 (69.2%) men	Peru	Lamivudine with either zidovudine (76% of cases), stavudine, or didanosine. Other drugs were nevirapine (*n* = 314 (57.5%)), efavirenz (*n* = 210 (38.5%)), lopinavir/ritonavir (*n* = 19 (3.5%)), atazanavir/ritonavir (*n* = 2 (0.4%)), or indinavir (*n* = 1 (0.2%))	Hepatotoxicity	7 (2.3%) cases	Not specified	[19]
			Rash	13 (2.4%) cases	Likely nevirapine or efavirenz	

765 patients: 614 (80.3%) men, 311 (40.6%) white, 265 (34.6%) black, 161 (21.0%) Hispanic	United States	Zidovudine, lamivudine, efavirenz (*n* = 380 (49.7%)). Zidovudine, lamivudine, abacavir, efavirenz (*n* = 373 (48.8%))	Grade 4 hepatotoxicity (ALT or/and AST, total bilirubin, direct bilirubin, alkaline phosphatase, *γ*-glutamyl transpeptidase raised >10 times over the upper limit of normal)	3 (4.3% of 70 patients who substituted efavirenz with nevirapine due to efavirenz toxicity) cases	Nevirapine	[20]
			Skin symptoms	18 (2.4%) cases 5 (33.3% of 15 patients who substituted efavirenz with nevirapine due to efavirenz toxicity) cases	Efavirenz Nevirapine	

103 pregnant women: 38 (36.9%) Caucasian, 24 (23.3%) Aboriginal	Canada	Nevirapine-based (*n* = 56 (54.4%))	Grade 4 hepatotoxicity (ALT or/and AST raised >10 times over the upper limit of normal)	1 (1.1% of 92 HAART-treated pregnancies) cases	Nevirapine	[21]
			Grade 3 hyperbilirubinemia (bilirubin raised 3–10 times over the upper limit of normal)	2 (2.2% of 92 HAART-treated pregnancies) cases	Likely nevirapine	
			Grade 2 rash and fever	2 (2.2% of 92 HAART-treated pregnancies) cases	Nevirapine	
			Grade 4 rash	1 (1.1% of 92 HAART-treated pregnancies) cases	Nevirapine	

137 patients, 103 (75.2%) men	Spain	Didanosine, stavudine, nelfinavir (*n* = 67 (48.9%)). Didanosine, stavudine, nevirapine (*n* = 70 (51.1%))	Hepatotoxicity		Nelfinavir Nevirapine	[22]
			Skin rash		Nelfinavir Nevirapine	
			GI ADRs (mainly diarrhea)	12 (14.9% of 67 nelfinavir patients and 2.8% of 70 nevirapine patients) cases	Nelfinavir Nevirapine	

573 patients, 366 (63.9%) men	Italy	Nevirapine-based 213 (37.2%) were taking zidovudine. 289 (50.4%) were taking stavudine. 71 (12.4%) were taking thymidine analogues. 97 (16.9%) were taking PIs	Hepatotoxicity (increases in serum liver function tests), including grade 3 hepatotoxicity (ALT or/and AST raised >5 times over the upper limit of normal if baseline levels were normal or >3 times over the baseline level if this was higher than the upper limit of normal)	44 (22.3% of 197 toxicity-related treatment interruptions) cases, including 5 cases of grade 3 AST elevations and 11 cases of grade 3 ALT elevations	Nevirapine and/or NRTIs	[23]
			Cutaneous reactions	84 (42.6% of 197 toxicity-related treatment interruptions) cases	Nevirapine	
			Unspecified GI ADRs	10 (5.1% of 197 toxicity-related treatment interruptions) cases	Nevirapine and/or NRTIs	

1318 patients, 967 (73.4%) men	Switzerland	Tenofovir, emtricitabine, atazanavir/ritonavir (*n* = 144 (10.9%)). Tenofovir, emtricitabine, efavirenz (*n* = 374 (28.4%)). Tenofovir, emtricitabine, lopinavir/ritonavir (*n* = 216 (16.4%)). Tenofovir, emtricitabine, nevirapine (*n* = 50 (3.8%)). Zidovudine, lamivudine, efavirenz (*n* = 77 (5.8%)). Zidovudine, lamivudine, lopinavir/ritonavir (*n* = 204 (15.5%)). Abacavir, lamivudine, efavirenz (*n* = 77 (5.8%))	Hepatic events	152 (11.5%) cases, including 42 (29.2%) of 144 atazanavir/ritonavir patients	Atazanavir/ritonavir (OR 2.55, 95% CI 1.01–6.42, *P* = 0.047) Other drugs	[24]
			HSR	38 (18.3% of 208 treatment interruptions due to drug toxicity) cases	Nevirapine (OR 3.33, 95% CI 1.43–7.77, *P* = 0.005) Atazanavir/ritonavir	
			GI tract intolerance	60 (28.9% of 208 treatment interruptions due to drug toxicity and 14.2% of 424 lopinavir/ritonavir patients) cases	Lopinavir/ritonavir (OR 5.50, 95% CI 2.67–11.3, *P* < 0.001)	

650 patients, 451 (69.4%) women	Botswana	Zidovudine/lamivudine, zidovudine/didanosine, or stavudine/lamivudine. 325 (50.0%) were taking nevirapine and 325 (50.0%) were taking efavirenz	Hepatotoxicity	11 (3.4% of 325 nevirapine patients) cases	Nevirapine	[25]
			Cutaneous HSR	19 (5.8% of 325 nevirapine patients) cases	Nevirapine	
			Pancreatitis	10 (3.3% of 325 nevirapine patients) cases	Likely nevirapine	

188 patients, 150 (79.8%) men	Spain	Efavirenz-based (*n* = 117 (62.2%)). Lopinavir/ritonavir-based (*n* = 71 (37.8%))	Hepatotoxicity	8 (5.1% of 117 efavirenz patients and 2.8% of 71 lopinavir/ritonavir patients) cases	Efavirenz Lopinavir/ritonavir	[26]
			Diarrhea	1 (0.9% of 117 efavirenz patients) case	Lopinavir/ritonavir Efavirenz	

2233 children	United States	Zidovudine, lamivudine (*n* = 1336 (59.8%)). Zidovudine, didanosine (*n* = 1022 (45.8%)). Stavudine, lamivudine (*n* = 1154 (51.7%)). Stavudine, didanosine (*n* = 772 (34.6%)). Didanosine, lamivudine (*n* = 258 (11.6%))	Hepatitis	56 (1.6% of 1336 zidovudine/lamivudine patients, 1.6% of 1022 zidovudine/didanosine patients, 1.6% of 1154 stavudine/lamivudine patients) cases	Zidovudine and lamivudine Zidovudine and didanosine Stavudine and lamivudine	[27]
			Bilirubin elevations	11 (0.5%) cases	Stavudine and lamivudine Stavudine and didanosine Lamivudine and didanosine Zidovudine and lamivudine Zidovudine and didanosine	
			Pancreatitis (assessed by routine monitoring of total amylase levels)	13 (1.7% of 772 stavudine/didanosine patients) cases	Stavudine and didanosine	

33 pregnant women: 20 (60.6%) Hispanic, 9 (27.3%) black, 4 (12.1%) white	United States	Zidovudine, lamivudine, nelfinavir	Grade ≤3 elevated AST and *γ*-glutamyl transpeptidase	2 (6.1%) cases	Treatment-related	[28]

289 patients: 204 (70.6%) men, 280 (96.9%) white	Spain	Nevirapine-based	Grade ≤3 elevated ALT/AST	6 (2.1%) cases	Nevirapine and hepatitis A virus coinfection	[29]

169 patients, 113 (66.9%) women	Cameroon	Zidovudine, lamivudine, nevirapine (*n* = 85 (50.3%)). Stavudine, lamivudine, nevirapine (*n* = 84 (49.7%))	Increases in ALT activity	1 (1.2% of 84 stavudine patients) case	Stavudine	[30]
			Rash	2 (1.2%) cases	Zidovudine and stavudine	

612 women: 443 (72.4%) black, 111 (18.1%) white, 53 (8.7) Hispanic	United States	Nevirapine-based (*n* = 152 (24.8%)). Nonnevirapine-based (*n* = 460 (75.2%))	Grade ≥2 liver enzyme elevations (graded according to the Toxicity Tables of the Division of Acquired Immunodeficiency Syndrome)	27 (4.4%) cases	Nevirapine and/or other drugs	[31]
			Grade ≥2 rash (graded according to the Toxicity Tables of the Division of Acquired Immunodeficiency Syndrome)	30 (5.7% of 526 patients included in the rash analysis and 26.3% of 114 patients who developed a new rash after therapy initiation) cases	Nevirapine and/or other drugs	
			SJS	1 (0.6% of 152 nevirapine patients)	Nevirapine	

88 children mean age 10.2 years: 51 (58.0%) girls, 38 (43.2%) white, 26 (29.5%) black	Switzerland	Lopinavir/ritonavir-based	AST elevation (185 IU/L)	1 (1.1%) case	Lopinavir/ritonavir	[32]
			HSR		Lopinavir/ritonavir	
			GI toxicity, including hepatic and pancreatic symptoms	5 (5.7%) cases	Lopinavir/ritonavir	
			Amylase elevation without serum lipase elevation (870 IU/mL)	1 (1.1%) case	Lopinavir/ritonavir	

883 patients, 606 (68.6%) men	Worldwide	Tenofovir, emtricitabine, atazanavir/ritonavir (*n* = 440 (49.8%)). Tenofovir, emtricitabine, lopinavir/ritonavir (*n* = 443 (50.2%))	Grade ≥3 increases in ALT/AST	25 (3.9% of 435 atazanavir/ritonavir patients and 1.8% of 431 lopinavir/ritonavir patients) cases	Atazanavir/ritonavir Lopinavir/ritonavir	[33]
			Grade ≥3 increases in total bilirubin levels	146 (33.6% of 435 atazanavir/ritonavir patients) cases	Mainly atazanavir/ ritonavir	
			Jaundice No mention of Gilbert syndrome or hemolysis	3 (0.7% of 440 atazanavir/ritonavir patients) cases	Atazanavir/ritonavir	
			Diarrhea and grade ≥2 nausea	4 (0.9% of 443 lopinavir/ritonavir patients) cases	Lopinavir/ritonavir	

40 patients, 20 (50.0%) women	Uganda	Zidovudine, didanosine, lopinavir/ritonavir (*n* = 36 (90.0%)). Stavudine, didanosine, lopinavir/ritonavir (*n* = 4 (10.0%))	Elevated AST levels	2 (5.0%) cases	Not specified	[34]
			Nausea or vomiting	7 (17.5%) cases	Didanosine and unspecified drugs	
			Diarrhea	9 (22.5%) cases	Not specified	

49 children, 30 (61.2%) boys	Burkina Faso	Didanosine, lamivudine, efavirenz	Increases in liver enzyme levels	2 (4.1%) cases	Likely didanosine	[35]
			Increases in pancreatic enzyme levels without pancreatitis	1 (2.0%) case	Likely didanosine	

3333 patients	England	Not specified	Jaundice No mention of Gilbert syndrome or hemolysis	7 (3.4% of 203 treatment switches) cases	Atazanavir	[36]
			Suspected/actual HSR	5 (2.5% of 203 treatment switches and 62.5% of 8 treatment switches due to abacavir toxicity) cases	Abacavir	
			Unspecified GI side effects	9 (4.4% of 203 treatment switches and 100% of 9 treatment switches due to saquinavir toxicity) cases	Saquinavir	
			Diarrhea	7 (3.4% of 203 treatment switches) cases	Lopinavir/ritonavir	

158 patients, 104 (65.8%) men	Italy	Tenofovir, emtricitabine, efavirenz (*n* = 41 (25.9%)). Tenofovir, emtricitabine, various boosted PIs (*n* = 46 (29.1%)). Abacavir, lamivudine, efavirenz (*n* = 12 (7.6%)). Abacavir, lamivudine, various boosted PIs (*n* = 41 (25.9%)). Other combinations including boosted PIs (*n* = 18 (11.4%))	Early HSR	2 (3.8% among 53 abacavir patients) cases	Abacavir	[37]

56 patients: 49 (87.5%) men, 26 (46.4%) white, 18 (32.1%) black	United States	Tenofovir, another NRTI, lopinavir/ritonavir, fosamprenavir (*n* = 28 (50.0%)). Tenofovir, other NRTIs, lopinavir/ritonavir (*n* = 14 (25.0%)). Tenofovir, other NRTIs, fosamprenavir/ritonavir (*n* = 14 (25.0%))	HSR		Abacavir	[38]

600 patients, 430 (71.7%) women	Uganda	Zidovudine, lamivudine plus either abacavir or nevirapine	Suspected HSR (grade ≤3)	15 (3.0% of 300 nevirapine patients and 2.0% of 300 abacavir patients) cases	Nevirapine Abacavir	[39]
			Suspected HSR (grade 4)	4 (1.3% of 300 nevirapine patients) cases	Nevirapine	

357 HLA B*5701-negative adults: 348 (97.5%) men, 307 (86%) white	Australia	Tenofovir and emtricitabine, or abacavir and lamivudine. Other drugs included zidovudine, didanosine, stavudine, atazanavir, lopinavir, efavirenz or nevirapine	HSR		Abacavir	[40]

385 HLA-B*5701-negative adults 313 (81.3%), men 56 (14.5%) black	Europe	Abacavir, lamivudine, efavirenz. Tenofovir, emtricitabine, efavirenz	HSR		Abacavir	[41]

211 children mean age 5 years: 111 (52.6%) boys	Zambia	Stavudine, lamivudine, nevirapine	Grade ≤2 rash	15 (7.1% of 211 nevirapine patients and 37.5% of 40 ADRs judged to be definitely/probably related to nevirapine) cases	Nevirapine	[42]

57 patients, 39 (68.4%) men	China	Stavudine, didanosine, nevirapine (*n* = 38 (66.7%)). Stavudine, lamivudine, nevirapine (*n* = 10 (17.5%)). Zidovudine, lamivudine, nevirapine (*n* = 9 (15.8%))	Rash (including grade 3 rash)	3 (5.3%) cases	Likely nevirapine	[43]

173 adults, 107 (61.8%) men	Cambodia	Efavirenz was substituted with nevirapine	Cutaneous HSR	10 (5.8% of 173 patients substituting efavirenz with nevirapine and 52.6% of 19 patients who developed nevirapine-induced treatment-limiting HSRs) cases	Nevirapine	[44]
			Hepatic HSR	10 (5.2% of 173 patients substituting efavirenz with nevirapine and 47.4% of 19 patients who developed nevirapine-induced treatment-limiting HSRs) cases	Nevirapine	

394 patients, 263 (66.8%) men	Cambodia	Stavudine, lamivudine, nevirapine	Minor rash	17 (4.3% of 394 patients who switched efavirenz with full-dose nevirapine and 32.7% of 52 cases of nevirapine-induced ADRs) cases 49 (7.4% of 661 ART-naive patients commencing nevirapine-based HAART and 51.6% of 95 cases of nevirapine-induced ADRs) cases	Nevirapine	[45]
			Severe HSR, including severe rash, SJS, TEN and/or hepatitis	35 (8.9% of 394 patients who switched efavirenz with full-dose nevirapine and 67.3% of 52 cases of nevirapine-induced ADRs) cases, including 30 cases of severe rash, 2 cases of SJS, and 3 cases of grade ≥3 hepatitis. 44 (6.6% of 661 ART-naive patients commencing nevirapine-based HAART and 46.3% of 95 cases of nevirapine-induced ADRs) cases, including 36 cases of severe rash (one fatal), 2 cases of SJS (one fatal), one case of fatal TEN, and 5 cases of grade ≥3 hepatitis	Nevirapine	
			Grade ≤4 hepatitis		Nevirapine	

121 adolescents mean age 7 years: 70 (57.8%) boys	Jamaica	77 (63.6%) receiving HAART Zidovudine/lamivudine-based (*n* = 72 (93.5%)). Nevirapine-based (*n* = 72 (93.5%)). Zidovudine, lamivudine, nevirapine (*n* = 67 (87.0%))	HSR	3 (4.2% of 72 nevirapine patients and 3.9% of 77 HAART patients) cases	Nevirapine	[46]
			Nausea and vomiting		Either zidovudine, lamivudine and/or nevirapine	

10186 patients: 7395 (72.6%) men, 6227 (61.1%) Caucasian	Europe and Canada	Nevirapine-based (*n* = 6547) Zidovudine, lamivudine, nevirapine (*n* = 4620 (45.4%))	Hepatotoxicity without concomitant skin rash	124 (1.9% of 4620 nevirapine patients and 27.1% of 458 patients who interrupted nevirapine due to HSRs) cases	Nevirapine	[47]
			Skin rash	334 (5.1% of 4620 nevirapine patients and 72.9% of 458 patients who interrupted nevirapine due to HSRs) cases	Nevirapine	
			Unspecified GI symptoms	402 (6.14% of 4620 nevirapine patients) cases	Nevirapine and/or NRTIs	
			Unspecified pancreas-related toxicities	4 cases among nevirapine patients	Nevirapine and/or NRTIs	

217 patients, 122 (56.2%) men	Senegal	Didanosine, lamivudine, efavirenz (*n* = 63 (29.0%)). Stavudine, didanosine, efavirenz (*n* = 44 (20.3%)). Stavudine, lamivudine, efavirenz (*n* = 11 (5.7%)). Zidovudine, lamivudine, efavirenz (*n* = 52 (24.0%)). Lamivudine, zidovudine, nevirapine (*n* = 28 (12.9%)). Didanosine, lamivudine, nevirapine (*n* = 8 (3.7%)). Stavudine, didanosine, nevirapine (*n* = 8 (3.7%)). Stavudine, lamivudine, nevirapine (*n* = 3 (1.4%))	Hepatitis, including hepatitis with concurrent skin rash	3 (6.4% of 47 nevirapine patients) cases, including 2 (4.2%) cases with concurrent skin rash	Nevirapine	[48]
			Skin rash, including SJS and TEN	3 (6.4% of 47 nevirapine patients) cases, including 2 (4.2%) cases of SJS or TEN	Nevirapine	
			Hyperamylasemia	10 (4.6%) cases	Likely efavirenz or nevirapine	

230 adults, 172 (74.8%) men	India	Stavudine, lamivudine, nevirapine (*n* = 157 (68.3%)). Stavudine, lamivudine, efavirenz (*n* = 18 (7.8%)). Zidovudine, lamivudine, nevirapine (*n* = 41 (17.8%)). Zidovudine, lamivudine, efavirenz (*n* = 14 (6.1%))	Severe rash (SJS or TEN)	9 (3.9%) cases, including 1 (0.4%) case of fatal TEN	Likely nevirapine or efavirenz	[49]

126 patients, 109 (86.5%) men	Spain and Italy	Lamivudine, abacavir, efavirenz (*n* = 63 (50.0%)). Lamivudine, abacavir, lopinavir/ritonavir (*n* = 63 (50.0%))	HSR/rash	8 (6.3%) cases	Efavirenz Lopinavir/ritonavir	[50]

21 Caucasian patients, 16 (76.2%) men	France	Efavirenz-based (*n* = 7 (33.3%)) Nevirapine-based (*n* = 14 (66.7%))	HSR	6 (28.6%) cases	Efavirenz Nevirapine	[51]

650 adults, 451 (69.4%) women	Botswana	Either zidovudine and lamivudine, zidovudine and didanosine, or stavudine and lamivudine, plus either nevirapine or efavirenz	SJS	16 (2.5%) cases	Likely nevirapine or efavirenz	[52]

66 patients, 56 (84.8%) men	Spain	Lopinavir/ritonavir-based (*n* = 33 (50.0%)). Nevirapine-based (*n* = 33 (50.0%)). NRTIs were didanosine, stavudine, and/or zidovudine	Diarrhea	10 (15.2%) cases	Likely nevirapine or lopinavir/ritonavir	[53]

70 patients, 50 (71.4%) men	Spain	Lopinavir/ritonavir-based	Unspecified GI symptoms assessed using the Gastrointestinal Symptom Rating Scale	1 (1.4%) case	Lopinavir/ritonavir	[54]

23 patients, 18 (78.3%) men	Spain	Zidovudine, lamivudine, abacavir, tenofovir	Unspecified GI symptoms		Lopinavir/ritonavir, tipranavir	[55]

115 patients, 70 (60.9%) men	France	Indinavir/ritonavir-based Lopinavir/ritonavir-based Nelfinavir-based	Unspecified GI ADRs	4 (12.5% among 32 lopinavir/ritonavir patients) cases	Lopinavir/Ritonavir	[56]
			Diarrhea	1 (3.2% among 32 nelfinavir patients) case	Nelfinavir	

1771 patients, 1204 (68.0%) men	South Africa	Zidovudine, didanosine, efavirenz (*n* = 444 (25.1%)). Stavudine, lamivudine, efavirenz (*n* = 444 (25.1%)). Zidovudine, didanosine, lopinavir/ritonavir (*n* = 440 (24.8%)). Stavudine, lamivudine, lopinavir/ritonavir (*n* = 443 (25.0%))	Nausea, constipation, fatigue		Zidovudine and didanosine. Stavudine and lamivudine	[57]

630 patients, 494 (78.4%) men	Worldwide	Saquinavir/ritonavir-based (*n* = 309 (49.0%)). Lopinavir/ritonavir-based (*n* = 163 (25.9%)). Indinavir/ritonavir-based (*n* = 158 (25.1%))	Unspecified GI toxicity, including grade ≥3 GI ADRs		Saquinavir	[58]

12 patients: 11 (91.7%) men, 9 (75.0%) Caucasian, 3 (25.0%) black	United Kingdom	Saquinavir/ritonavir-based	Mild nausea and diarrhea	5 (41.7%) cases	Likely treatment-related	[59]

1081 patients, 708 (65.5%) men	Italy	Not specified	Pancreatic toxicity (at least 3-fold increases in serum pancreatic enzymes)	166 (38.2% of 435 patients with confirmed laboratory pancreatic abnormalities) cases	Concurrent use of didanosine, stavudine, lamivudine	[60]

## Abbreviations

ADR: Adverse drug reaction
ALT: Alanine aminotransferase
ART: Antiretroviral therapy
AST: Aspartate aminotransferase
CI: Confidence interval
CYP: Cytochrome p450
GI: Gastrointestinal
HAART: Highly active antiretroviral therapy
HCV: Hepatitis C virus
HIV: Human immunodeficiency virus
HLA: Human leukocyte antigen
HSR: Hypersensitivity syndrome reaction
IQR: Interquartile range
NRTI: Nucleoside reverse transcriptase inhibitor
NNRTI: Non-nucleoside reverse transcriptase inhibitor
OR: Odds ratio
PI: Protease inhibitor
SJS: Stevens-Johnson syndrome
UGT: Uridine diphosphate glucuronosyltransferase.
